# Intrageneric cross-reactivity of monospecific rabbit antisera against venoms of the medically most important *Naja* spp. African snakes

**DOI:** 10.1371/journal.pntd.0011545

**Published:** 2023-08-15

**Authors:** Aarón Gómez, Andrés Sánchez, Gina Durán, Mauren Villalta, Álvaro Segura, Mariángela Vargas, Daniela Solano, María Herrera, Melvin Sánchez, José María Gutiérrez, Guillermo León

**Affiliations:** Instituto Clodomiro Picado, Facultad de Microbiología, Universidad de Costa Rica, San José, Costa Rica; Fundação de Medicina Tropical Doutor Heitor Vieira Dourado, BRAZIL

## Abstract

**Background:**

Envenomations by African snakes represent a high burden in the sub-Sahara region. The design and fabrication of polyspecific antivenoms with a broader effectiveness, specially tailored for its use in sub-Saharan Africa, require a better understanding of the immunological features of different *Naja* spp. venoms of highest medical impact in Africa; and to select the most appropriate antigen combinations to generate antivenoms of wider neutralizing scope.

**Methodology/principal findings:**

Rabbit-derived monospecific antisera were raised against the venoms of five spitting cobras and six non-spitting cobras. The effects of immunization in the animal model were assessed, as well as the development of antibody titers, as proved by immunochemical assays and neutralization of lethal, phospholipase A_2_ and dermonecrotic activities. By the end of the immunization schedule, the immunized rabbits showed normal values of all hematological parameters, and no muscle tissue damage was evidenced, although alterations in aspartate aminotransferase (AST) and alkaline phosphatase (ALP) suggested a degree of hepatic damage caused mainly by spitting cobra venoms. Immunologic analyses revealed a considerable extent of cross-reactivity of monospecific antisera against heterologous venoms within the spitting and no-spitting cobras, yet some antisera showed more extensive cross-reactivity than others. The antisera with the widest coverage were those of anti-*Naja ashei* and anti-*N*. *nigricollis* for the spitting cobras, and anti-*N*. *haje* and anti-*N*. *senegalensis* for the non-spitting cobras.

**Conclusions/significance:**

The methods and study design followed provide a rationale for the selection of the best combination of venoms for generating antivenoms of high cross-reactivity against cobra venoms in sub-Saharan Africa. Results suggest that venoms from *N*. *ashei*, *N*. *nigricollis* within the spitting cobras, and *N*. *haje* and *N*. *senegalensis* within the non-spitting cobras, generate antisera with a broader cross-reactivity. These experimental results should be translated to larger animal models used in antivenom elaboration to assess whether these predictions are reproduced.

## Author summary

The African elapids pose a major potential to induce clinically important envenomations and severe sequelae after a snakebite accident. Within the genus *Naja*, different radiation events have occurred rendering the African cobras with diverse features in their venom composition. Commonly divided into spitting and non-spitting cobras, their venom is characterized as either cytotoxic or neurotoxic, respectively, based on the pathophysiology of envenomation. In this study, an experimental protocol was developed based on the generation of monospecific antisera in rabbits immunized with venoms of spitting and non-spitting African cobras. Cross-reactivity of monospecific antisera was assessed by immunochemical analyses and by neutralization of the lethal, phospholipase A_2_ (PLA_2_) and dermonecrotic activities. Results showed a large extent of intrageneric cross-reactivity by all antisera, yet few exceptions were noticed. The venoms that generated antisera with the highest cross-neutralization ability among the spitting cobras were *Naja nigricollis* and *N*. *ashei*, whereas for the non-spitting cobras were *N*. *haje* and *N*. *senegalensis*. These results provide evidence for the selection of the best combination of immunogens for antivenom production with the widest neutralization scope within the African cobras. Additionally, the results obtained in the rabbit model should be translated to a larger animal model used for antivenom fabrication to assess whether these predictions are confirmed.

## Introduction

The African snakes with the major potential to induce clinically relevant envenomation in humans belong to the Lamprophiidae, Colubridae, Elapidae, and Viperidae families [1–2]. In turn, the most important elapids belong to the genera *Dendroaspis* (mambas), *Hemachatus* (Rinkhals), *Naja* (cobras), and *Pseudohaje* (false cobras) [2]. Within the genus *Naja* different adaptation events have occurred, which might have been triggered by ecological and geological events associated with the formation and expansion of the African Rift Valley. Furthermore, within the spitting cobras, at least three events have rendered the African spitting cobras as a diverse clade (i.e., the appearance and expansion of the grassland and large mammals, the volcanic activity in the western Branch of the East African Rift System, and the presence of past arid corridors from northeastern to southwestern Africa). It is likely that the spitting venom trait developed before this radiation [3].

According to the reptile database (https://reptile-database.reptarium.cz/), the *Naja* genus comprises 23 African species, which are commonly classified as spitting or non-spitting cobras, depending on their ability to project a venom spray from their fangs. Moreover, on the basis of the clinical characteristics of envenomations, spitting and non-spitting cobras are also classified as cytotoxic and neurotoxic cobras, respectively.

Only five spitting cobras, i.e., *N*. *ashei* (Ashe’s spitting cobra), *N*. *katiensis* (Mali cobra), *N*. *mossambica* (Mozambique spitting cobra), *N*. *nigricincta* (Barred spitting cobra) and *N*. *nigricollis* (Black-necked spitting cobra), and six non-spitting cobras, i.e., *N*. *anchietae* (Anchieta’s cobra), *N*. *annulifera* (Snouted cobra), *N*. *haje* (Egyptian cobra), *N*. *melanoleuca* (Central African forest cobra), *N*. *nivea* (Cape cobra), and *N*. *senegalensis* (Senegalese cobra) are associated with high levels of morbidity, disability, or mortality, and therefore considered by WHO as Category 1 species [2].

Cobra venoms are mainly composed of three-finger toxins (3FTxs), phospholipases A_2_ (PLA_2_s), snake venom metalloproteinases (SVMPs), cysteine-rich secretory proteins (CRISPs), and other less abundant components such as L-amino acid oxidase (LAAO), cobra venom factor (CVF), venom nerve growth factor (vNGF), and vascular endothelial growth factor (VEGF), whose relative abundance in the venom cocktail (i.e., as relative concentration percentage) varies inter- and intra-specifically [4–11]. In general, the proteins that predominate in the venoms of African cobras are 3FTxs and PLA_2_s, which are largely responsible for the toxicity of these venoms.

Spitting cobras have venom which, when instilled in the eyes, induces ophthalmia, blepharitis, and blindness [12,13], whereas intradermal or subcutaneous injection induces severe local necrosis, without neurotoxic manifestations [14,15]. Loss of function due to chronic ulceration, osteomyelitis, arthrodesis, hypertrophic or keloid scars, and tetanus are frequent complications of necrotic lesions produced by spitting cobras [15,16]. After several years, some scars can undergo malignant transformation to produce what is known as Marjolin’s ulcer [15].

On the other hand, neurotoxic cobras have venoms that induce progressive muscular paralysis that could involve respiratory muscles resulting in respiratory arrest and death [5,15,17]. Thus, observation and management of local tissue damage and pro- inflammatory actions (i.e., mostly caused by the abundance of cytotoxins), should be monitored along with neurotoxicity, which could lead to paralysis [5,9].

In cases of cobra envenomation, the recommended treatment is the intravenous administration of specific snake antivenoms, i.e., formulations of whole immunoglobulins, or their Fab or F(ab´)_2_ fragments, purified from plasma of animals immunized towards snake venoms [2]. Since the identification of the offending snake species is not always possible and the envenomation symptoms could overlap between different species, the use of polyspecific antivenoms with wide neutralization scope is usually recommended [15,18].

Nevertheless, their efficacy is limited to venoms antigenically similar to those used as immunogens to stimulate the immune response in animals used to manufacture the antivenom [5,9,18–21]. Hence, to ensure a wide coverage of neutralization without requiring the identification of the offending snake species, the use of polyspecific formulations is preferred [2,18].

The selection of venoms used as immunogen to produce polyspecific antivenoms is based on the variation/conservation of the antigenic characteristics of venoms of the medically most important snakes in the region where the antivenom is intended to be used. The selection of the most appropriate venoms for immunization should, therefore, be based on a detailed knowledge of the antigenic relatedness of venoms and the medical relevance of the species [2,5,9,18–21]. This represents a challenge in African cobra venoms, due to the abundance, diversity, and wide distribution of species [3].

In this work, we used a rabbit model to determine the antigenic relatedness between venoms of African cobra snakes classified by WHO as Category 1 species, based on the intra-species cross-reactivity between monospecific rabbit sera raised against individual spitting and non-spitting cobra venoms. This information could be useful for the rational, knowledge-based design of the most appropriate venom mixtures for the generation of pan-African antivenoms.

## Materials and methods

### Ethics statement

This work presents an experimental study conducted following the standard procedures of scientific ethics, including those relating to the use and care of animals. All procedures conducted in this study meet the International Guiding Principles for Biomedical Research Involving Animals [22]. All procedures involving animals were approved by the Institutional Committee for the Care and Use of Laboratory Animals of Universidad de Costa Rica (approval code CICUA 202–2020). Rabbits and mice of both sexes were obtained from the Bioterium of Clodomiro Picado Institute. Rabbits were managed in Scanbur type EC3 cages (L 823 * W 660 * H 110 mm), one rabbit per cage, while mice were handled in Tecniplast Eurostandard Type II 1264C cages (L 25 * W 40 * H 14 cm), five mice per cage. In both cases, animals were maintained at 18–24°C, 60–65% relative humidity, and a 12:12 h light-dark cycle.

### Snake venoms

Venoms of adult specimens of *N*. *anchietae* (from Namibia, batch #527.002), *N*. *annulifera* (from Mozambique, batch #622.040), *N*. *ashei* (from Kenya, batch #410.191), *N*. *haje* (unknown origin, batch #222.061), *N*. *katiensis* (from Burkina Faso, batch #705.010), *N*. *melanoleuca* (unknown origin, batch #516.031), *N*. *mossambica* (from Tanzania, batch #627.002), *N*. *nigricincta* (from South Africa, batch #507.081), *N*. *nigricollis* (unknown origin, batch #616.031), *N*. *nivea* (from South Africa, batch #524.010), and *N*. *senegalensis* (from Mali, batch #805.010) were purchased from Latoxan (Portes-dès Valence, France). After collection, venom was stabilized by lyophilization and stored at -40°C. Solutions of venom were prepared immediately before use.

### Reverse-phase HPLC profiling

Five milligrams of each venom were dissolved in 200 μL of 0.1% trifluoroacetic acid (TFA) and 5% acetonitrile buffer (buffer A). Insoluble material was removed by centrifugation and the proteins in the supernatant were separated by reverse-phase HPLC (RP-HPLC, HPLC system: Agilent 1100 series; Agilent Technologies), equipped with a C18 column (250 x 4.6 mm, 5 μm particle size; Agilent Technologies). The flow rate was set to 1 mL/min and the protein separation was performed with the following buffer gradient: 0% buffer B (buffer B: 95% acetonitrile, 0.1% TFA) for 5 min, followed by 0–15% B over 10 min, 15–45% B over 60 min, 45–70% B over 10 min and 70% B for 9 min [23]. Protein peaks were detected at 215 nm. The similarity in the HPLC fractions were identified by comparing the chromatograms with those previously published.

### Determination of lethal activity

Groups of five mice (16–18 g; CD-1 strain; both sexes) received a subcutaneous (SC) administration of the analgesic Tramadol, at a dose of 50 mg/kg, to reduce pain during the test [24]. Fifteen minutes afterwards, mice received an intraperitoneal (IP) injection of 0.5 mL of 0.12 M NaCl, 0.04 M phosphate, pH 7.2 solution (PBS) containing different amounts of venom. The number of deaths during the following 6 h were recorded [25] and used to estimate the Median Lethal Dose (LD_50_; i.e., the amount of venom that results in death of 50% of the injected mice) by Probits [26]. Surviving mice were euthanized by CO_2_ inhalation. Results were reported as LD_50_ and the corresponding 95% confidence interval (95% CI).

### Determination of phospholipase A_2_ activity

The phospholipase A_2_ (PLA_2_) activity was determined by the methodology described by Gutiérrez and coworkers [27]. Briefly, various venom doses were prepared in duplicate for each venom, and then, 100 μL of each solution was added to 1 mL of egg yolk diluted 1:5 in a solution of 0.1 M Tris, 0.01 M CaCl_2_ and 1% Triton X-100 (pH 8.5). The mixtures were incubated at 37°C for 30 min. Finally, the free fatty acids were extracted and titrated according to Dole [28]. The activity was expressed as μEq of fatty acid released/mg venom/minute.

### Determination of dermonecrotic activity

Dermonecrotic activity was assessed as described by Rivel et al. [29]. Various amounts of venom, dissolved in 100 μL PBS, were injected by the intradermal route into groups of three mice (both sexes, CD-1, 18-20g). Injections were done in the ventral abdominal region. After 72 h, animals were euthanized by CO_2_ inhalation and the diameter of lesions on the inner surface of the abdominal skin was determined. The Minimum Necrotizing Dose (MND) was estimated by linear regression as the amount of venom that induces a lesion of 5 mm diameter.

### Immunization of rabbits

Groups of four rabbits (both sexes, New Zealand, 2.5–3.0 kg body weight) were immunized with the venoms of single species of either spitting cobras (i.e., *N*. *ashei*, *N*. *katiensis*, *N*. *mossambica*, *N*. *nigricincta* or *N*. *nigricollis*), or non-spitting cobras (i.e., *N*. *anchietae*, *N*. *annulifera*, *N*. *haje*, *N*. *melanoleuca*, *N*. *nivea* or *N*. *senegalensis*). Immunization was performed by five SC injections, applied at two-week intervals. Total volume of injections was 2 mL and all of them contained 1 mg of venom. In the first injection, the venoms were emulsified in Complete Freund’s Adjuvant (CFA); in the second one, Incomplete Freund’s Adjuvant (IFA) was used; afterwards, in the rest of the immunization protocol, venoms were dissolved in PBS. Rabbits were monitored during immunization by a veterinarian to observe signs of toxicity. At the end of immunization, samples of 6 mL blood were collected from the ear marginal vein; 3 mL were added to EDTA as anticoagulant and used for hematological tests, while 3 mL were left to clot in order to obtain serum, which was used for serum chemistry and immunological tests. Immediately after the collection of blood, rabbits were euthanized by an overdose of anesthetic (i.e., a dose of 100 mg/kg of sodium pentobarbital, administered by the IP route).

### Hematological and serum chemistry analysis

Hematological and serum chemistry analyses were conducted on each individual rabbit sample. Hematological analyses (i.e., erythrocyte, leukocyte and platelet counts, hematocrit, and hemoglobin concentration) were conducted in a Veterinary Hematology Analyzer (Exigo Eos Hematology System; Boule Diagnostics AB, Stockholm, Sweden). The following analytes were quantified in a clinical chemistry analyzer (Spin200E Automatic biochemistry analyzer; Spinreact, Barcelona, España): creatine kinase (CK), alanine aminotransferase (ALT), aspartate transaminase (AST), and alkaline phosphatase (ALP) were determined by the corresponding International Federation of Clinical Chemistry and Laboratory Medicine (IFCC) methods. Urea was quantified by a modification of the Talke and Schubert method [30]; creatinine by a kinetic modification of the Jaffe colorimetric method [31]; albumin by the bromocresol green colorimetric method [32]; and total protein by the Biuret method [33].

### Immune reactivity of rabbit sera by enzyme-linked immunosorbent assay (ELISA)

Polystyrene plates (Costar 9017; 96 well; flat bottom) were coated overnight at room temperature with 100 μL of PBS containing 3 μg of venom. After washing the plates five times with distilled water, 100 μL of several dilutions (from 1:1,500 to 1:40,500, dilution factor 3) of each rabbit serum sample, in PBS-2% bovine serum albumin (BSA), were added. Plates were incubated for 1 h at room temperature and washed five times. Afterwards, 100 μL of goat anti-rabbit IgG conjugated with peroxidase (Sigma-Aldrich A0545), diluted 1:5000 with PBS-2% BSA, were added to each well. Microplates were incubated for 1 h at room temperature. After a final washing step, color was developed by the addition of H_2_O_2_ and *o*-phenylenediamine (2 mg/mL *o*-phenylenediamine, 1 μL/mL hydrogen peroxide in 0.1M citrate buffer, pH 5.0). Color development was stopped by the addition of 1.0 M HCl. Absorbances at 492 nm were recorded. The relative concentration of anti-venom antibodies in the samples was calculated by interpolation of their absorbances in a calibration curve. Relative concentration was expressed as percentage, 100% corresponding to the titer of the serum raised against the homologous venom of each species. Results were expressed as mean ± SD of all rabbits in each group.

### Electrophoretic analysis and western blot

Venoms (30 μg) were separated by SDS-PAGE run under non-reducing conditions using an acrylamide concentration of 12% [34]. Molecular weight markers (3 μL) were included into the gels (Thermo 26630). The gels were either stained with Coomassie Brilliant Blue R-250, decolored with water and used to display the electrophoretic profile of the venoms or transferred to a nitrocellulose membrane at 30 mAmp during 1 h. The gels used for the Western blot were later stained using Coomassie Brilliant Blue R-250 to confirm the protein transfer to the membranes. Then, the membranes were blocked with PBS-0.1% casein for 30 min. Next, membranes were incubated for 1 h with a pool of serum samples of all rabbits of each monospecific antiserum, diluted 1/500 with PBS-0.1% casein. After washing the membranes four times with PBS-0.1% casein, they were incubated for 1 h with goat anti-rabbit IgG conjugated with alkaline phosphatase, diluted 1:2000 with PBS-0.1% casein. Finally, after the last washing step, 5-bromo-4-chloro-3-indolyl-phosphate/nitroblue tetrazolium (BCIP/NBT) color development substrate (Sigma-Aldrich) was added, and the reaction was stopped with distilled water.

### Neutralization of the lethal, PLA_2_, and dermonecrotic activities

The ability of pools of serum samples from all rabbits in each group to neutralize the activities of the venoms was assessed by mixing a constant challenge dose of each venom with different dilutions of the pool of each antiserum. Mixtures were incubated at 37°C for 30 min before determining the residual activity of venom by using the experimental systems described above. The challenge doses utilized were: 2 LD_50_s for lethal activity, for PLA_2_ activity a challenge dose of 3 μg for the spitting cobras and a range of 1.5–75 μg for the non-spitting cobras, and one MND for the dermonecrotic activity. In all cases, venom-only controls were included, in which venom was incubated with PBS instead of antiserum. The use of 2 LD50s as a challenge dose in the neutralization of lethality studies, instead of the usual 4–5 LD_50_s, is justified to increase the sensitivity of the assay, favoring the detection of cross-reactivity of antisera against venoms. For lethal activity, neutralization was expressed as the Median Effective Dose (ED_50_), defined as the ratio mg venom/mL antiserum at which the activity of venom was reduced to 50% [35]. For PLA_2_ and dermonecrotic activities, neutralization was expressed as Median Effective Dose (ED_50_), defined as the ratio of venom/antivenom in which the activity of venom was reduced by 50% [11]. For comparing the different antisera in terms of their ability to neutralize the effects of the lethal and dermonecrotic activities of the heterologous venoms, a value of 1 was given to antisera that had an equal or higher value of ED_50_ as compared to homologous antisera, and a value of 0 was assigned to antisera having a lower value of ED_50_ as compared to homologous antiserum. Thus, a ranked score was created assigning a summatory value to each of the antisera assessed.

### Statistical analyses

In the case of lethality and its neutralization, groups having non-overlapping values of 95% CI were considered significantly different. For ELISA, PLA_2_ and dermonecrotic activities, the significance of the differences between mean values of groups was assessed by one-way ANOVA, followed by a Ryan-Einot-Gabriel-Welsch Range (R-E-G-W Q) in order to test all pairs of means by conforming subsets which are significantly different between them and, at the same time, excluding variables from one group to another (i.e., *p*-value >0.05 is considered for grouping subsets). Additionally, R-E-G-W Q test poses a good estimation power and a tight control over the Type I Error. The interpretation of this test should be addressed as variables that conform homogenous subgroups that exclude other groups of variables (i.e., either venom or antisera) at *p*-values >0.05 in a comparison of all pairs of means. The hematological and serum chemistry variables were assessed by one-way ANOVA, followed by a Dunnett’s t post-hoc test in order to test the pairs of means compared to a specified control group (i.e., *p*-value <0.05 was considered significant). Neutralization of the PLA_2_ and dermonecrotic activities was assessed by a Kruskal-Wallis nonparametric test. Linearity and homogeneity of variances were tested, and a *p*-value <0.05 was considered significant, except when otherwise specified.

## Results and discussion

### General characterization of *Naja* spp. venoms

The proteomic analyses of venoms of *N*. *annulifera* [6,8,9], *N*. *ashei* [7], *N*. *haje* [4,10], *N*. *katiensis* [10,36], *N*. *melanoleuca* [5], *N*. *mossambica* [8, 36, 37], *N*. *nigricincta* [37], *N*. *nigricollis* [10,36,38], *N*. *nivea* [21], and *N*. *senegalensis* [20] have been previously described. However, to the best of our knowledge, the complete proteomic or venom profiling analysis of *N*. *anchietae* venom has not been conducted even though this species poses a medical threat.

**Fig 1:**
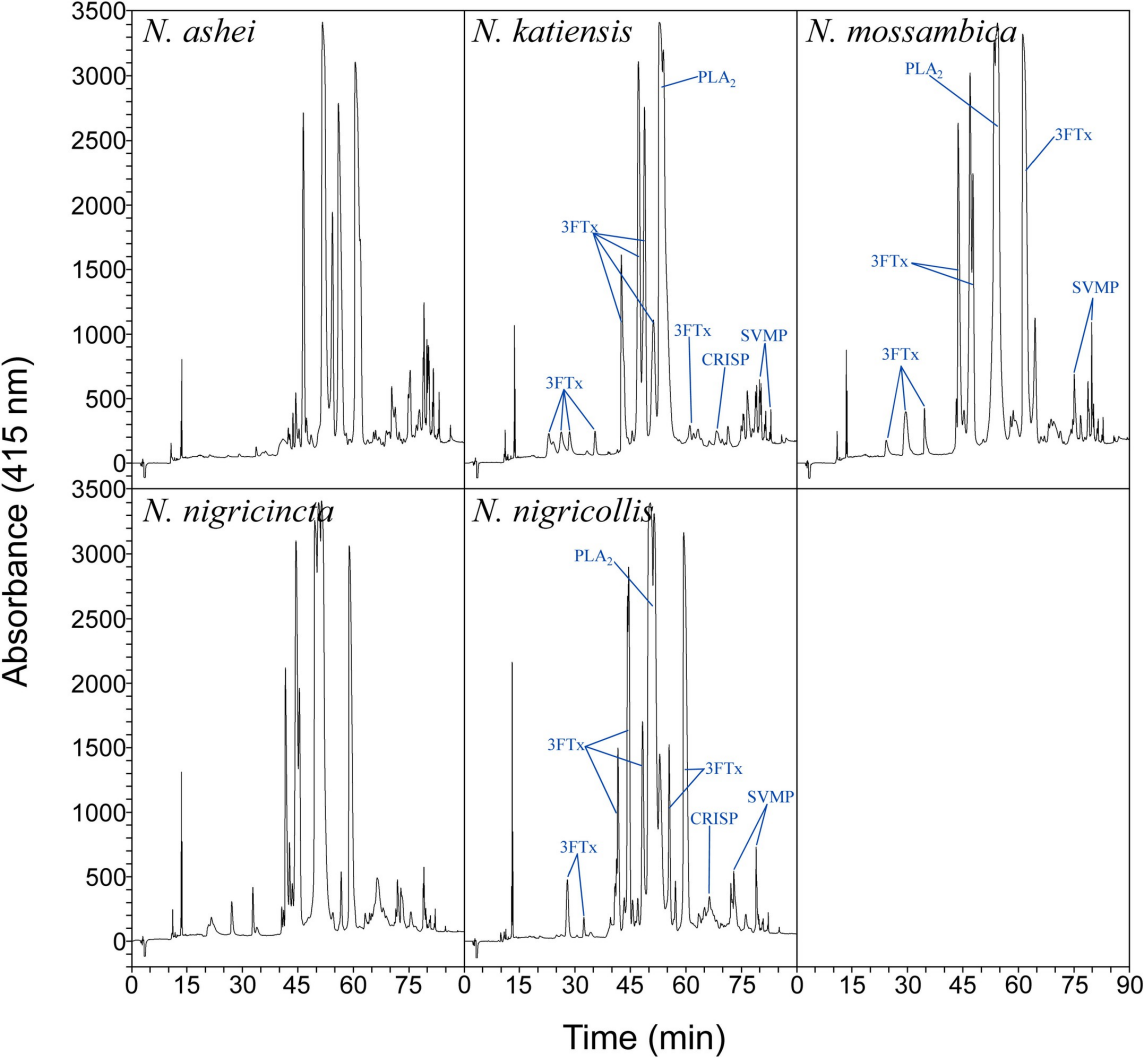
RP-HPLC chromatograms of spitting cobra venoms. Labeling of peaks was done based on Petras et al. [36] for the profiles of *N*. *katiensis*, *N*. *mossambica* and *N*. *nigricollis*.

**Fig 2:**
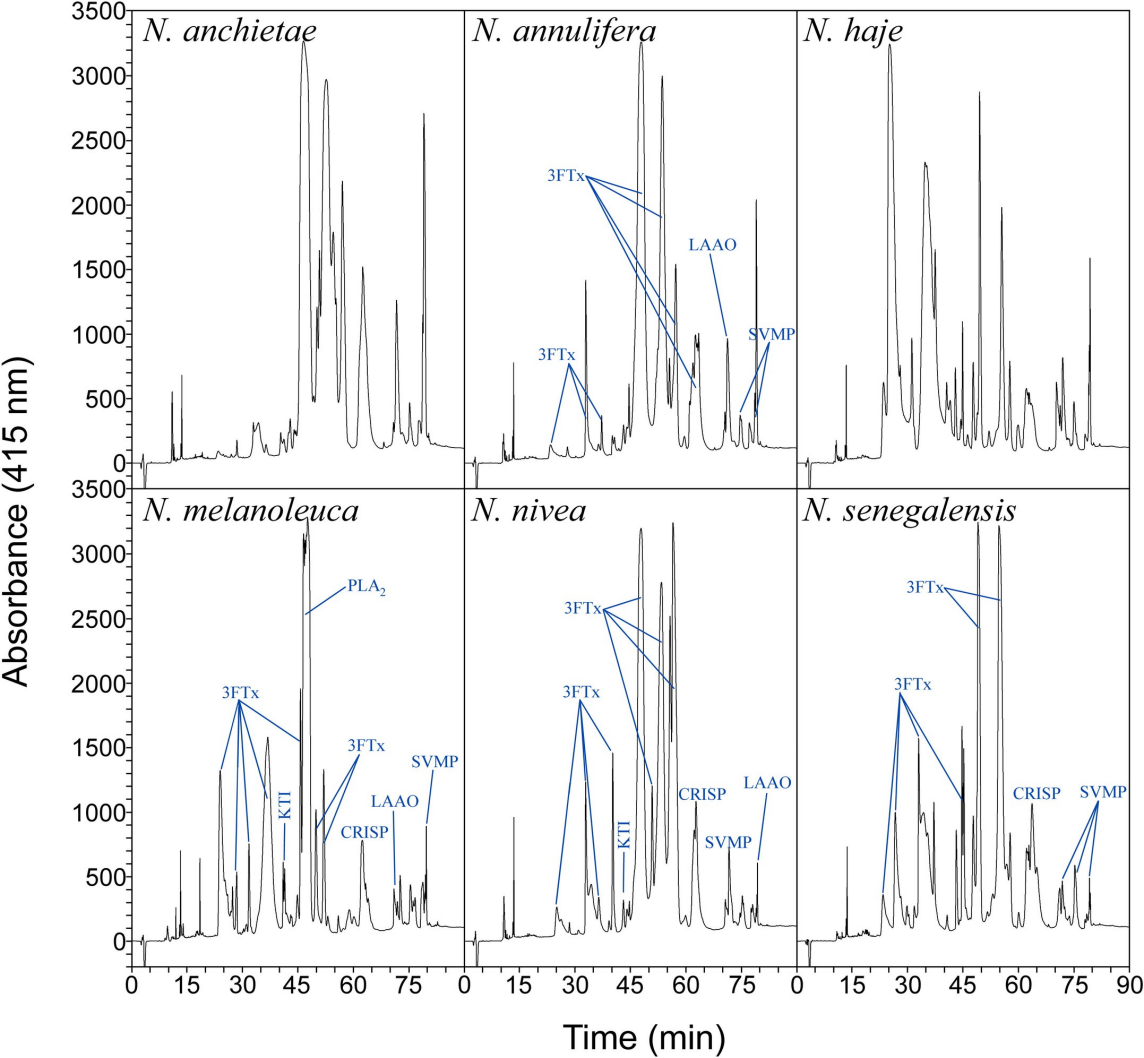
RP-HPLC chromatograms of non-spitting cobra venoms. Labeling of peaks was done based on Tan et al. [9] for *N*. *annulifera*, Lauridsen et al. [5] for *N*. *melanoleuca*, Tan et al. [21] for *N*. *nivea*, and Wong et al. [20] for *N*. *senegalensis* profiles.

The chromatographic venom profiles of the spitting cobras (Fig 1 and S2 Data) and non-spitting cobras (Fig 2 and S3 Data) were obtained. As expected, there are intra-species variations in the relative abundance of various fractions, compared to previously published data. Nonetheless, a pattern of higher abundance of the fractions corresponding to cytotoxins and neurotoxins (i.e., 3FTxs) and PLA_2_s can be inferred for the spitting and non-spitting cobra venoms (S4 Data). In the case of spitting cobra venoms, cytotoxic 3FTxs and PLA_2_s predominate in their proteome [36] and are likely to be responsible for lethality.

The venoms of spitting cobras had similar LD_50_s, i.e., overlapping 95% CI, whereas the venoms of the non-spitting cobras showed differences with a low LD_50_ for *N*. *melanoleuca* and *N*. *senegalensis*, and even lower LD_50_ value for *N*. *haje*, the latter being the most toxic venom (Table 1 and S1 Data). The lower toxicity of venoms of the spitting cobras compared to those of some non-spitting cobras can be due to a different composition and relative concentration of neurotoxic 3FTxs and PLA_2_s [5,10,20,21]. The venoms of *N*. *haje*, *N*. *melanoleuca* and *N*. *senegalensis*, which have the highest toxicity, are rich in neurotoxic 3FTxs [5,10,20]. The high toxicity demonstrated by *N*. *haje* venom is associated to a severe neurotoxic syndrome, evidenced by limb paralysis, and labored respiration in mice, mainly due to the abundance of neurotoxic 3FTxs in its composition [4,10]. Toxicovenomics analysis aimed at identifying the toxins mainly responsible for lethality in these venoms is a pending task.

**Table 1:** Toxic activities of venoms of African Cobra snakes.

Venom	Lethality (LD_50_)^a^	Phospholipase (PLA_2_)^b^	Dermonecrotic (MND)^c^
**Spitting Cobras**			
*N*. *ashei*	21.7 (15.8–30.5)	158 ± 2	18 ± 1
*N*. *katiensis*	18.9 (13.7–24.8)	176 ± 2	29 ± 6
*N*. *mossambica*	23.0 (16.1–32.6)	156 ± 15	26 ± 1
*N*. *nigricincta*	21.7 (15.8–30.5)	154 ± 2	14 ± 1
*N*. *nigricollis*	18.4 (10.7–26.4)	149 ± 2	12 ± 1
**Non-spitting Cobras**			
*N*. *anchietae*	69.7 (47.3–95.6)	5 ± 0	ND*
*N*. *annulifera*	63.5 (48.5–84.6)	4 ± 0	ND*
*N*. *haje*	1.9 (1.4–2.7)	6 ± 1	ND*
*N*. *melanoleuca*	6.4 (4.9–8.9)	342 ± 16	ND*
*N*. *nivea*	22.4 (17.8–28.3)	4 ± 2	ND*
*N*. *senegalensis*	7.4 (5.7–10.1)	7 ± 1	ND*

^a^ Lethality is expressed as LD_50_ (95% CI) by the i.p. route, i.e., the Median Lethal Dose, defined as the amount of venom (μg) that results in the death of 50% of injected mice.

^b^ Phospholipase A_2_ activity is expressed as μEq of fatty acid released per mg protein per min. PLA_2_ activity was determined using 3 μg of venom for the spitting cobras and a range of 1.5–75 μg for the non-spitting cobras (see Materials and Methods for details). Results are shown as mean ± SD (n = 3).

^c^ Dermonecrotic activity is expressed as Minimum Necrotizing Dose (MND, μg/mouse, n = 3). Results are shown as mean ± SD.

*ND: No dermonecrotic activity was detected at the highest venom doses tested (20 μg for *N*. *haje*, *N*. *melanoleuca* and *N*. *senegalensis* venoms, and 80 μg for *N*. *anchietae* and *N*. *annulifera* venoms. Statistical analyses of PLA_2_ and MND activities are detailed in the text.

The spitting cobra venoms showed a lower PLA_2_ activity compared to previous works [11,36], yet no differences were observed among the five species studied (F_(4; 5)_ = 3.963, *p* = 0.82). *N*. *katiensis* venom showed high PLA_2_ activity even though its PLA_2_ content is lower than that of *N*. *nigricollis* venom [10] (Table 1 and S1 Data).

The non-spitting cobra venoms had a lower PLA_2_ activity when compared to the spitting cobras (F_(10; 11)_ = 340.400, *p*< 0.001). Moreover, differences among the six non-spitting cobra species were seen (F_(5; 6)_ = 435.968, *p*< 0.001), with activity of *N*. *melanoleuca* venom being considerable higher than the rest (R-E-G-W Q post-hoc test *p* = 1.0) of the non-spitting cobra venoms studied. As a pattern among *Naja* sp venoms, PLA_2_ is the second most abundant protein family [5,10,11]. Regarding non-spitting cobras, the higher content of PLA_2_ in the venom of *N*. *melanoleuca* [5], compared to *N*. *annulifera* [9], *N*. *nivea* [21], and *N*. *senegalensis* [20] venoms, may explain the difference found in this enzymatic activity.

Only the spitting cobras induced dermonecrotic lesions in mice (Table 1 and S1 Data). The dermonecrotic activity of these venoms varied significantly (F_(4; 7)_ = 17.982, *p*< 0.001), with the venom of *N*. *nigricincta* and *N*. *nigricollis* showing the highest activity (R-E-G-W Q post-hoc test *p* = 0.596). Since the major constituents of spitting cobra venoms are 3FTxs and PLA_2_, it has been demonstrated that some of these PLA_2_s induce cytotoxicity [39] and they may have a role in the dermonecrotic effect in murine models. Likewise, a large number of 3FTxs exhibit general cytolytic effects, being referred to as cytotoxins, causing a distinctive severe local tissue damage and necrosis without clear evidence of neurotoxicity [10,14,29,36].

Overall, the composition of the venom of spitting cobras is consistent with this toxicological profile and the high content of cytotoxic 3FTxs and PLA_2_s are likely responsible for the tissue necrosis that characterizes these envenomations. Spitting cobra venom contains 7–10% of SVMPs [10,11,36]. However, it is unlikely that they play a role in the pathogenesis of dermonecrosis [29]. On the other hand, despite the presence of cytotoxins and PLA_2_s in non-spitting cobra venoms, they did not induce necrosis in mice (Table 1 and S1 Data), suggesting that 3FTxs and PLA_2_s in these venoms are likely to exert a neurotoxic effect instead of cytotoxicity.

Envenomations from spitting and non-spitting cobras differ from a biogeographical standpoint [3] revealing, with the aid of proteomics and transcriptomics, significant variations in venom composition [10,36,37]. Particularly important are the variations in the cytotoxic effect between the spitting and non-spitting cobras, suggesting an alternative evolutionary path for venoms that, biologically, could provide a predominantly defensive role as well as the use of distinct niches [40]. Additionally, these differences in cobra venom phenotypes, as a result of multiple environmental and genetic interactions [8] and different behavioral responses such as predation and defensive actions (i.e., spitting, and non-spitting defensive mechanisms), may influence the clinical outcome in human envenomations.

Envenomations by African *Naja* sp present with a broad range of clinical manifestations, from necrosis and tissue damage to hematotoxicity and paralysis [8,15]. Different clinical presentations are the basis for the syndromic approach used in Africa to diagnose the type of envenomation and to select the appropriate antivenoms for treatment [15].

### Effects induced by *Naja* spp. venoms in immunized rabbits

Groups of rabbits were immunized with venoms of spitting and non-spitting cobras. The first two immunization injections were administered in a water/oil emulsion (i.e., Freund’s Complete Adjuvant and Freund’s Incomplete Adjuvant), from which venoms are slowly released, reducing the tissue damage caused by the venom and enhancing the antibody response of the animals. Assuming that the antibody response developed during the initial two immunizations could reduce the toxic effects of the additional venom boosters, and to minimize the inflammation caused by Freund’s adjuvants, in the rest of the immunization scheme venoms were dissolved in PBS. Thus, it is of interest to evaluate the venom-induced toxic effects in this animal model.

**Fig 3:**
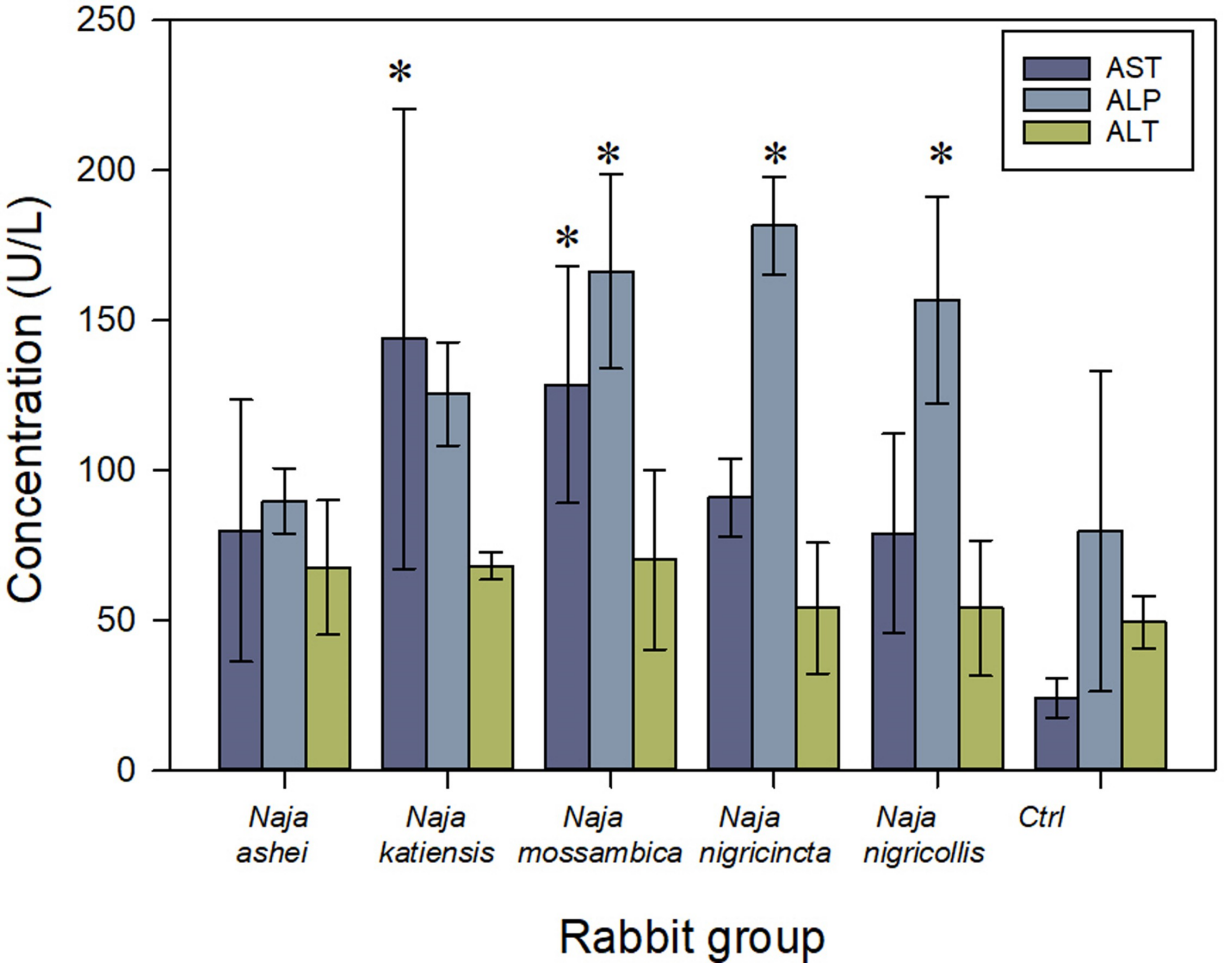
Activity of aspartate aminotransferase (AST), alanine aminotransferase (ALT), and alkaline phosphatase (ALP) in serum of rabbits immunized with the venoms the spitting cobras *Naja ashei*, *N*. *katiensis*, *N*. *mossambica*, *N*. *nigricincta* and *N*. *nigricollis*. Results are expressed in International Units (IU) per L and correspond to the mean ± SD of the four rabbits in each group. * *p*< 0.05 when compared to rabbit control groups.

During immunization, local inflammatory reactions, at the site of injection of adjuvants and venom, were observed. By the end of the immunization schedule, the immunized rabbits showed normal values of all hematological parameters analyzed [41] (S1 Table). In addition, no muscle tissue damage was evidenced during the immunization since the CK plasma levels were within the range of the control, non-immunized rabbits [41] (S2 Table). However, the spitting cobra venoms caused some alterations in the plasma levels of AST (F_(5; 18)_ = 4.025, *p* = 0.013) and ALP (F_(5; 18)_ = 7.299, *p*< 0.001), but not in the plasma levels of ALT (Fig 3), when compared to control rabbits [41,42]. These alterations were specially marked in the group of rabbits immunized with *N*. *katiensis* and *N*. *mossambica* venoms (Dunnett’s t-test *p* = 0.003; *p* = 0.011, respectively) for the AST, and *N*. *mossambica*, *N*. *nigricincta* and *N*. *nigricollis* venoms (Dunnett’s t-test *p* = 0.004; *p*< 0.001; *p* = 0.010, respectively) for the ALP. These differences in the AST, but mainly in the ALP levels, suggest a degree of hepatic damage caused by the venom of spitting cobras during immunization. In agreement, there has been clinical evidence of hepatic damage in patients bitten by *N*. *nigricollis* [43]

Since cytotoxicity and myotoxicity are generally caused by the action of PLA_2_s and cytotoxic 3FTxs on several cell targets, the use of cytotoxin and PLA_2_ inhibitors during the immunization scheme could be an option to reduce tissue damage, a hypothesis to be evaluated in large animal models used for antivenom production.

In contrast, the non-spitting cobra venoms did not cause significant alterations in the plasma levels of the analytes studied compared with the non-immunized control group [41,42] (S2 Table). However, the plasma chemistry tests used do not detect neurotoxic alterations; therefore, a different approach should be considered when assessing the effects of the immunization scheme for non-spitting cobra venoms and other neurotoxic venoms.

Additionally, normal values of creatinine and urea in serum were found in all rabbits [41,42] (S2 Table) except for the group immunized with *N*. *melanoleuca* and *N*. *senegalensis* venoms, where the urea value was significantly different from the control group (Dunnett’s t-test *p* = 0.017). Likewise, normal values of total protein concentration in serum were found in all rabbits [41,42] (S2 Table) except for the groups immunized with *N*. *anchietae* and *N*. *annulifera*, where the total protein value was significantly different from the control group (Dunnett’s t-test *p* = 0.032; *p* = 0.028, respectively), suggesting that a hydric stress could have developed in these rabbits. Taking into consideration the plasma biochemistry results, it is concluded that no significant kidney injury was induced during immunization.

Our findings highlight the need to conduct analysis of local tissue and liver damage when venoms of spitting and non-spitting cobras are used in larger animal models for antivenom production. Additionally, strategies evaluating the neurotoxicity in larger animal models should be introduced, and the use of cytotoxin and PLA_2_ inhibitors could be added to the immunization mixture in order to minimize the extent of these pathophysiological manifestations.

### Cross-reactivity and neutralization between spitting cobra venoms

**Fig 4:**
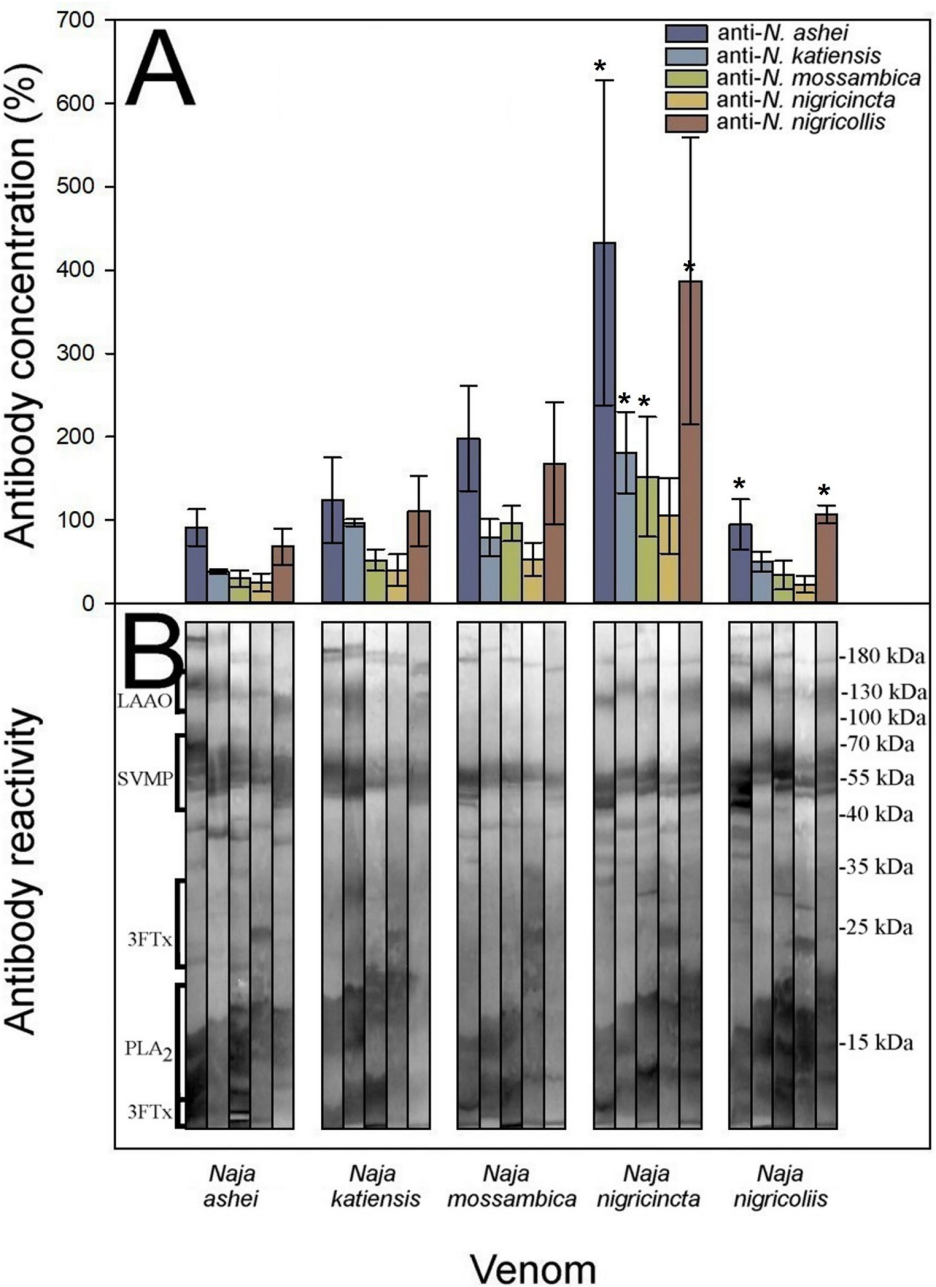
Cross-reactivity between the spitting cobra venoms was determined by **A**) ELISA and **B**) Western blot. ELISA results are expressed as percentage considering as 100% the titer of serum raised against the homologous venom of each species and correspond to the mean ± SD of all rabbits in each group. Values with * are significantly different when compared to homologous antisera according to R-E-G-W Q post-hoc test.

We followed the experimental protocol described by Gómez and colleagues [44] for African viperid venoms, which combines antibody titers quantified by ELISA, Western Blot, and the neutralization of toxic activities, allowing the assessment of the intrageneric cross-reactivity between monospecific rabbit sera against homologous and heterologous venoms. Cross-reactivity was noticed among all anti-*Naja* antisera against the spitting cobra venoms, revealing a clear antigenic venom similarity, yet there were quantitative differences between them (Fig 4 and S1 Data).

Unexpectedly, in the spitting cobras the highest antibody responses not always were observed against the homologous venoms (Fig 4A), and all the monospecific antisera showed cross-reactivity by ELISA against the heterologous venoms (anti-*N*. *ashei* F_(4; 15)_ = 8.543, *p* = 0.001; anti-*N*. *katiensis* F_(4; 15)_ = 6.147, *p* = 0.004; anti-*N*. *mossambica* F_(4; 15)_ = 6.551, *p* = 0.003; anti-*N*. *nigricincta* F_(4; 15)_ = 4.218, *p* = 0.017; anti-*N*. *nigricollis* F_(4; 15)_ = 5.451, *p* = 0.006). The anti-*N*. *nigricincta* antiserum showed a weak cross-reactivity against the heterologous venoms, yet the *N*. *nigricincta* antigen showed the highest antibody recognition by the heterologous anti-*Naja* antisera (R-E-G-W Q post-hoc test anti-*N*. *ashei p* = 1.0; anti-*N*. *katiensis p* = 1.0; anti-*N*. *mossambica p* = 0.956; anti-*N*. *nigricollis p* = 1.0).

The venom of *N*. *nigricollis* was recognized by the heterologous anti-*N*. *ashei* antiserum with a similar antibody titer of its homologous antiserum (R-E-G-W Q post-hoc test *p* = 0.944). The antisera against *N*. *ashei* and *N*. *nigricollis* showed the highest cross-reactivity against all the venoms tested (F_(4; 15)_ = 8.543, *p* = 0.001; F_(4; 15)_ = 5.451, *p* = 0.006, respectively; Fig 4A).

Extensive cross-reactivity was also detected by Western blot (Fig 4B). The identification of the immunoreactive protein bands was based on the molecular masses as shown in Fig 4B. Moreover, in concordance with the ELISA results, the stronger reactions seen by Western blot varied against the homologous antigens, with a similar scenario for the heterologous antigens such as SVMPs, 3FTxs, and PLA_2_s protein families (Fig 4B).

**Fig 5:**
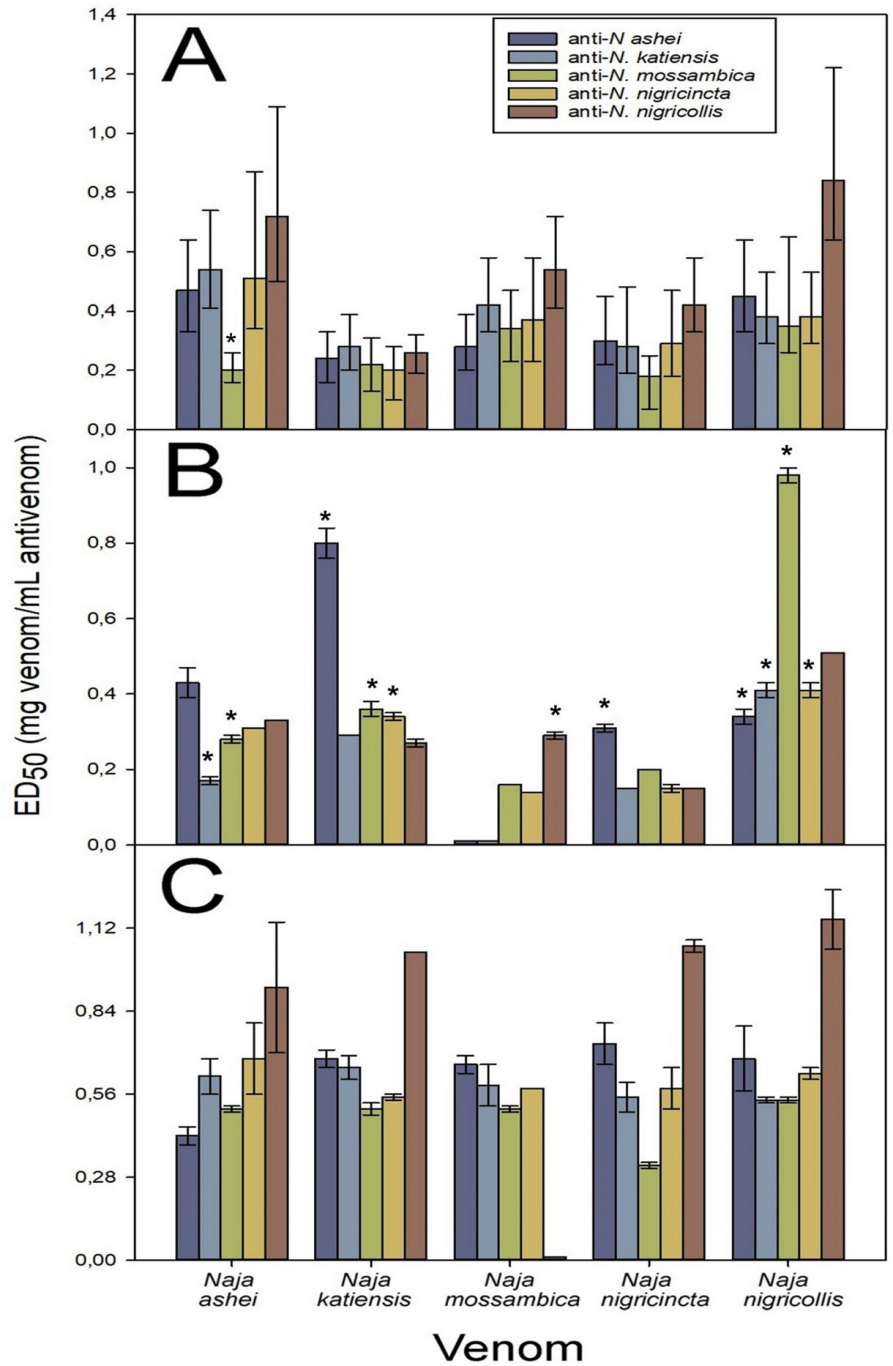
Cross-neutralization of the spitting cobra venom toxic effects by the antisera. **A**) Neutralization of the lethal activity, **B**) Neutralization of the phospholipase A_2_ activity, **C**) Neutralization of the dermonecrotic activity. In the case of lethality neutralization, error bars represent the 95% CI and non-overlapping values with homologous antisera are depicted by *. In the case of PLA_2_ neutralization, error bars represent SD and * depict values that were significantly different from homologous antisera; while in the case of dermonecrotic neutralization, error bars represent SD.

When the cross-neutralization of the spitting cobra venom toxic effects was analyzed, a wide neutralization of lethality by all the homologous and heterologous antisera was observed, with varying ED_50_ values but with a high extent of overlapping in the 95% CI (Fig 5A and S1 Data). Additionally, a general trend was observed in that the anti-*N*. *nigricollis* antiserum showed the highest neutralization efficacy against the heterologous venoms, and the anti-*N*. *mossambica* antiserum showed a lower neutralization efficacy against the heterologous venoms, although these differences were mostly non-significant.

Regarding the neutralization of the PLA_2_ activity, with few exceptions the antisera neutralized the homologous and heterologous venoms, albeit with different ED_50_ values (Kruskal-Wallis test *N*. *ashei*: *p* = 0.071; *N*. *katiensis*: *p* = 0.064; *N*. *mossambica*: *p* = 0.068; *N*. *nigricincta*: *p* = 0.065; *N*. *nigricollis*: *p* = 0.065). As a general trend, the anti-*N*. *ashei* and the anti-*N*. *nigricollis* antisera showed a good performance in neutralizing the PLA_2_ activity of the heterologous venoms when compared to the homologous antisera and, to a lesser extent, the anti-*N*. *katiensis* and the anti-*N*. *nigricincta* antiserum showed a similar behavior (Fig 5B and S1 Data). The anti-*N*. *ashei* and anti-*N*. *katiensis* antisera were unable to neutralize PLA_2_ activity of *N*. *mossambica* venom. The dermonecrotic activity of the spitting cobras was neutralized by the homologous and heterologous antisera with ED_50_ values that did not differ significantly between them (Fig 5C and S1 Data) (Kruskal-Wallis test *N*. *ashei*: *p* = 0.243; *N*. *katiensis*: *p* = 0.320; *N*. *mossambica*: *p* = 0.139; *N*. *nigricincta*: *p* = 0.308; *N*. *nigricollis*: *p* = 0.103). Despite the lack of significant difference, there is a trend of the anti-*N*. *nigricollis* antiserum to have a high neutralization of the dermonecrotic activity of the heterologous venoms when compared to the homologous antisera, except for *N*. *mossambica* venom where no neutralization was observed (Fig 5C). This venom was well neutralized by the rest of monospecific antivenoms.

Taken into consideration the data gathered from the neutralization of lethality and dermonecrosis, which in the case of spitting cobra venoms is a clinically relevant effect, the anti-spitting cobras’ antisera can be scored from highest to lowest cross-reactivity score (Table 2) as follows: anti-*N*. *ashei*, anti-*N*. *nigricollis*, anti-*N*. *katiensis*, anti-*N*. *nigricincta*, and anti-*N*. *mossambica*.

**Table 2:** Ranking of the antisera performance based on the neutralization capacity of the lethal and dermonecrotic activities of spitting and the lethal activity of the non-spitting cobra venoms. A score system was used assigning a value of 1 to the heterologous antisera whose neutralizing capacity was equal or higher than that of the homologous antisera, and a value of 0 when the neutralizing capacity was lower than that of the homologous antiserum.

	Spitting Cobra venoms														
	ED_50_ Lethal Activity							ED_50_ Dermonecrotic Activity							
**Antiserum**	*N*. *ashei*	*N*. *katiensis*		*N*. *mossambica*	*N*. *nigricincta*		*N*. *nigricollis*	*N*. *ashei*	*N*. *katiensis*		*N*. *mossambica*	*N*. *nigricincta*		*N*. *nigricollis*	**Total score**
Anti-N. ashei	-	1		1	1		1	-	1		1	1		0	**7**
Anti-N. katiensis	1	-		1	1		0	1	-		1	1		0	**6**
Anti-N. mossambica	0	1		-	1		1	1	0		-	0		0	**4**
Anti-N. nigricincta	1	1		1	-		0	1	0		1	-		0	**5**
Anti-N. nigricollis	1	1		1	1		-	1	1		0	1		-	**7**
	**Non-spitting Cobra venoms**														
	**ED**_**50**_ **Lethal Activity**														
**Antiserum**	*N*. *anchietae*		*N*. *annulifera*			*N*. *haje*		*N*. *melanoleuca*		*N*. *nivea*			*N*. *senegalensis*		**Total score**
Anti-N. anchietae	-		1			0		1		1			0		**3**
Anti-N. annulifera	1		-			0		0		0			0		**1**
Anti-N. haje	1		1			-		1		1			1		**5**
Anti-N. melanoleuca	1		1			0		-		1			0		**3**
Anti-N. nivea	1		1			0		0		-			0		**2**
Anti-N. senegalensis	1		1			0		1		1			-		**4**

Finally, the extensive cross-reactivity of the monospecific antisera generated in this work agrees with previous studies where antivenoms produced using different mixtures of *Naja* spp. venoms were able to neutralize the lethal, PLA_2_, and dermonecrotic effects of these venoms [5,9,11,19–21,36,45,46]. According to our findings, the venoms of *N*. *ashei* and *N*. *nigricollis* were well neutralized by the spitting cobras’ heterologous antisera, whereas the anti-*N*. *ashei* and the anti-*N*. *nigricollis* antisera had a better overall neutralizing capacity among the heterologous antisera compared to the other antisera, with the exception of dermonecrotic activity of *N*. *mossambica* venom (Table 2).

### Cross-reactivity and neutralization between non-spitting cobra venoms

**Fig 6:**
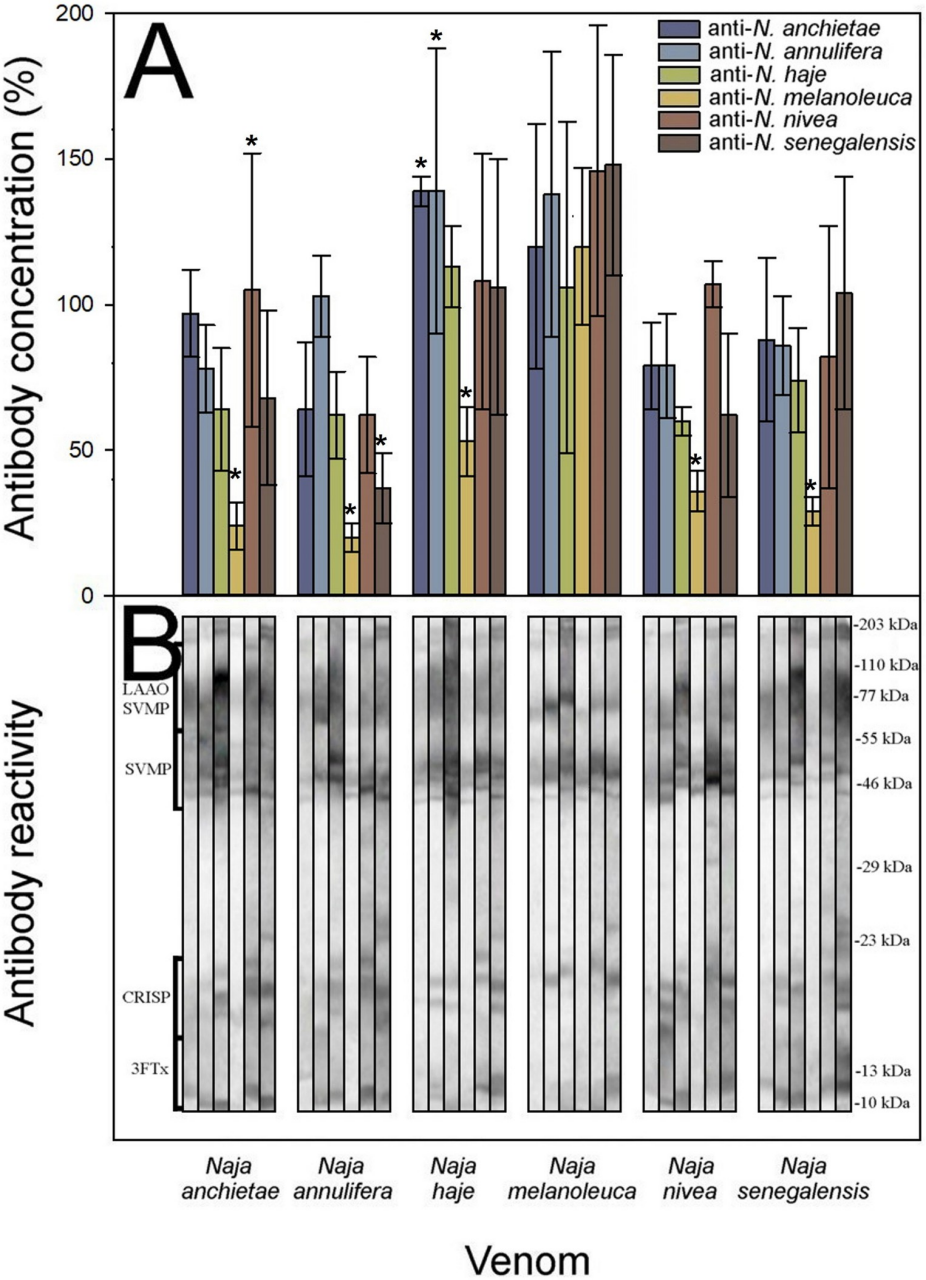
Cross-reactivity between the non-spitting cobra venoms was determined by **A**) ELISA and **B**) Western blot. ELISA results are expressed as percentage considering as 100% the titer of serum raised against the homologous venom of each species and correspond to the mean ± SD of all rabbits in each group. Values with * are significantly different when compared to homologous antisera according to R-E-G-W Q post-hoc test.

The monospecific non-spitting cobras antisera responded differently in terms of its cross-reactivity quantified by ELISA (Fig 6A and S1 Data). The venoms of *N*. *anchietae* (F_(5; 15)_ = 4.814, *p* = 0.008), *N*. *annulifera* (F_(5; 15)_ = 13.985, *p*< 0.001), *N*. *haje* (F_(5; 15)_ = 3.168, *p* = 0.038), *N*. *nivea* (F_(5; 15)_ = 6.973, *p* = 0.001), and *N*. *senegalensis* (F_(5; 15)_ = 3.216, *p* = 0.036) were recognized to a different extent by the homologous and heterologous antisera. The venom of *N*. *melanoleuca* (F_(5; 14)_ = 0.437, *p* = 0.815) did not show significant differences in its cross-reactivity when assessed with either homologous or heterologous antisera (Fig 6A).

*N*. *anchietae* venom was poorly recognized by the anti-*N*. *melanoleuca* antiserum (R-E-G-W Q post-hoc test *p* = 0.051), whereas the anti-*N*. *nivea* antiserum showed a higher cross-reactivity when compared to the homologous anti-*N*. *anchietae* antiserum (R-E-G-W Q post-hoc test *p* = 0.302). Likewise, the venom of *N*. *annulifera* was poorly recognized by the anti-*N*. *melanoleuca* and anti-*N*. *senegalensis* antisera when compared to the homologous anti-*N*. *annulifera* antiserum (R-E-G-W Q post-hoc test *p* = 0.351).

The venom of *N*. *haje* was better recognized by the anti-*N*. *anchietae* and anti-*N*. *annulifera* antisera when compared to the homologous anti-*N*. *haje* antiserum (R-E-G-W Q post-hoc test *p* = 0.680), whilst the anti-*N*. *melanoleuca* antiserum cross-reacted poorly with *N*. *haje* venom (R-E-G-W Q post-hoc test *p* = 0.249) when compared to the homologous antiserum. The venom of *N*. *melanoleuca* was recognized to a similar extent by the heterologous antisera when compared to the homologous antiserum (R-E-G-W Q post-hoc test *p* = 0.852).

*N*. *nivea* venom showed a low cross-reactivity with the heterologous antisera, especially in the case of anti-*N*. *melanoleuca* antiserum (R-E-G-W Q post-hoc test *p* = 0.126) when compared to the homologous anti-*N*. *nivea* antiserum. A similar scenario was evidenced for *N*. *senegalensis* venom, with a low cross-reactivity by the heterologous antisera, especially in the case of anti-*N*. *melanoleuca* antiserum (R-E-G-W Q post-hoc test *p* = 0.083) when compared to the homologous antiserum. Moreover, the anti-*N*. *melanoleuca* antiserum recognized to a lesser extent *N*. *anchietae* (R-E-G-W Q post-hoc test *p* = 0.051), and *N*. *annulifera* (R-E-G-W Q post-hoc test *p* = 0.351) venoms.

Cross-reactivity was also detected by Western blot, and the identification of the immunoreactive protein bands, based on the molecular masses is shown in Fig 6B. Furthermore, in agreement with the ELISA results, the strongest reactions by Western blot were observed with heterologous antisera in some cases, with a similar scenario for heterologous antigens such as SVMP, 3FTx, PLA_2_ and LAAO protein families. Noteworthy, higher reactivity was observed in high molecular mass bands (SVMPs, LAAOs), as compared to the bands corresponding to PLA_2_s and 3FTxs. Nevertheless, a wide variation in the intensity of the immune recognition by homologous and heterologous antisera against the antigens was observed by Western blot (Fig 6B).

**Fig 7:**
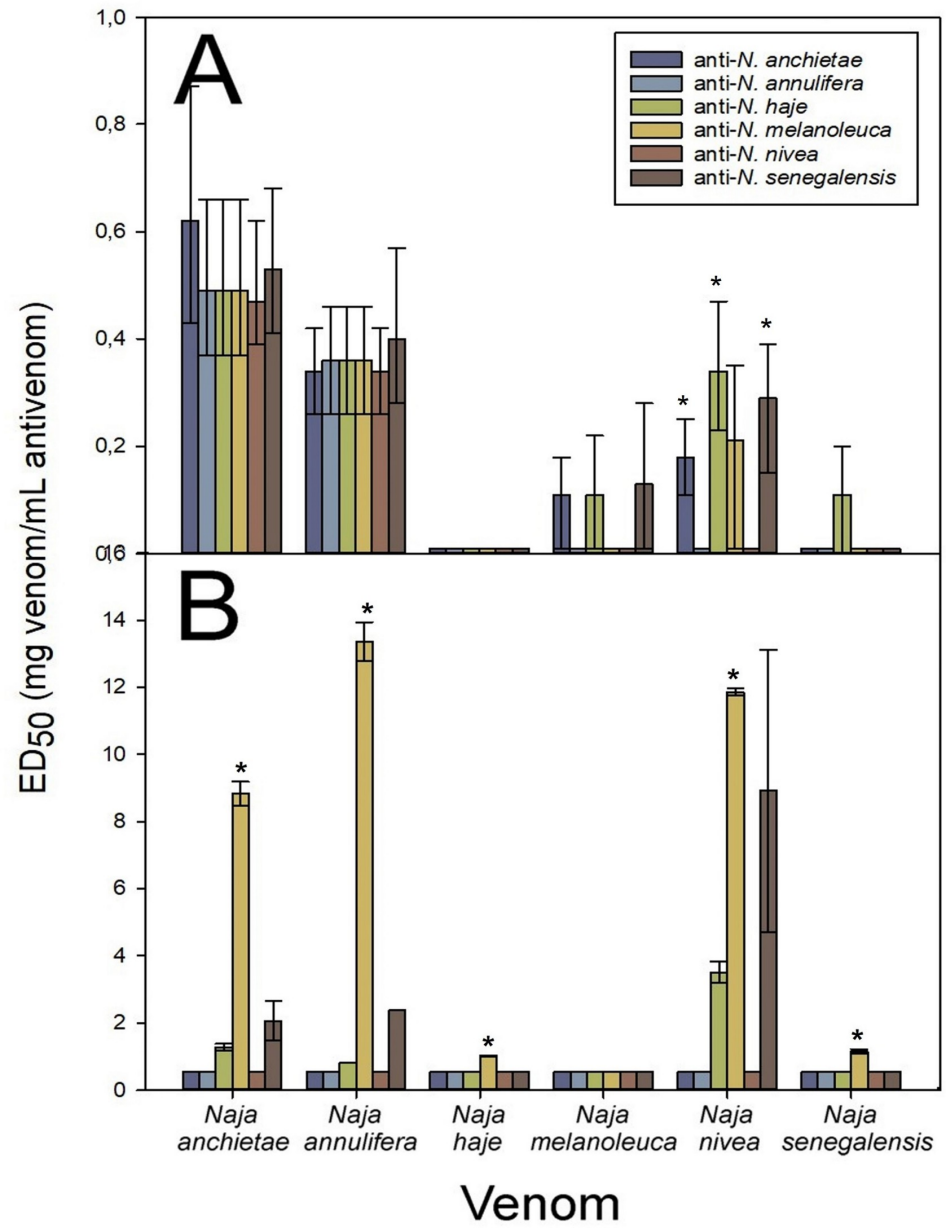
Cross-neutralization of the non/spitting cobra venom toxic effects by the antisera. **A**) Neutralization of the lethal activity, **B**) Neutralization of the phospholipase A_2_ activity. In the lethal activity neutralization, error bars represent the 95% CI and non-overlapping values are depicted by *. In the case of PLA_2_ activity, error bars represent SD and * depict values that were significantly different from homologous antisera.

When the cross-neutralization of venoms of non-spitting cobra toxic effects was analyzed, there were variations in the values of ED_50_ regarding neutralization of lethality, with a high extent of overlapping in the 95% CI (Fig 7A and S1 Data). Particularly, the anti-*N*. *haje* antiserum showed a good neutralization profile of the heterologous non-spitting cobra venoms. However, the venom of *N*. *haje* was poorly neutralized by either the homologous or heterologous antisera (Fig 7A). Likewise, the venom of *N*. *senegalensis* was poorly neutralized by the antisera, with the exception of *N*. *haje* antiserum.

The neutralization of PLA_2_ activity by the non-spitting cobra antisera showed variable ED_50_ values for the homologous and heterologous venoms (Kruskal-Wallis test *N*. *anchietae p* = 0.054; *N*. *annulifera p* = 0.053; *N*. *haje p* = 0.053; *N*. *nivea p* = 0.060, *N*. *senegalensis p* = 0.053). The anti-*N*. *melanoleuca* serum showed the best PLA_2_ neutralization (Kruskal-Wallis test *p*< 0.001) except for the homologous venom (Fig 7B and S1 Data), with an overall good neutralizing capacity against PLA_2_ activity of the heterologous venoms. The venoms of *N*. *haje*, *N*. *melanoleuca* and *N*. *senegalensis* were poorly neutralized by the homologous and heterologous antisera.

Recently, a low content of PLA_2_ in the venom *N*. *haje* [4], and the incapability of detecting PLA_2_ in the venoms of *N*. *annulifera* [9], *N*. *nivea* [21] and *N*. *senegalensis* [20] has been described, while PLA_2_s comprise 13% of the proteome *N*. *melanoleuca* venom [5]. This may explain to some extent the high PLA_2_ activity of and the anti-PLA_2_ response elicited by this venom.

Considering together the data gathered from neutralization of lethality, the anti-non-spitting cobras’ antisera can be graded from highest to lowest cross-reactivity score (Table 2) as follows: anti-*N*. *haje*, anti-*N*. *senegalensis*, anti-*N*. *anchietae*, anti-*N*. *melanoleuca*, anti-*N*. *nivea*, anti-*N*. *annulifera*.

## Conclusions

As a general conclusion, antisera anti-*N*. *ashei* and anti-*N*. *nigricollis* provided the highest immune coverage based on the score obtained from the lethality and dermonecrosis ED_50_ values for spitting cobra venoms, whereas antiserum anti-*N*. *haje* provided the highest immune coverage based on the ranked score obtained from the lethality ED_50_ values for non-spitting cobra venoms. Nonetheless, it is likely that the generation of antivenoms with the broadest neutralization scope will require the inclusion of venoms from additional *Naja* species, a hypothesis that will be assessed in future studies.

The cross-reactivity seen between monospecific anti-spitting cobra antisera suggests antigenic similarities between the toxic components of *N*. *ashei*, *N*. *katiensis*, *N*. *mossambica*, *N*. *nigricincta* and *N*. *nigricollis* venoms. Likewise, with some exceptions, cross-reactivity between monospecific anti-non-spitting cobra antisera suggests a degree of similarity between the toxic components of *N*. *anchietae*, *N*. *annulifera*, *N*. *melanoleuca*, and *N*. *nivea* venoms. However, *N*. *haje*, *N*. *melanoleuca* and *N*. *senegalensis* venoms are poorly neutralized by heterologous antisera. Insights into the basis of this phenomenon demand high-throughput technologies, such as antivenomics and toxicovenomics [5,11,47], to determine antigenic similarities between venoms and to identify the toxins responsible for overall toxicity [47].

Finally, as immunogenicity depends on the nature of the animal’s immune system selected as immunoglobulin source, the conclusions of our experiments in rabbits cannot be directly extrapolated to animal models used in antivenom production, such as horses or sheep. However, our observations can be applied to formulate rational hypotheses that can be put to experimental evaluation by immunizing horses with mixtures of several cobra venoms to produce broad neutralizing antivenoms for sub-Saharan Africa.

## Supporting information

S1 Table Hematological parameters of rabbit groups immunized with the venoms of the spitting cobras. (DOCX)

S2 Table Biochemical parameters of rabbit groups immunized with spitting and non-spitting cobra venoms. Values are compared to the Control group and range reference values are provided. Results are presented as mean ± SD. (DOCX)

S1 Data Raw data from the immunoreactivity assays (i.e., ELISAs), phospholipase A_2_ (PLA_2_) activity, dermonecrotic (MND) activity, lethal activity (LD_50_s), and neutralization (ED_50_s) of lethal activity, PLA_2_ and MND for the spitting and non-spitting cobra venoms assessed. (XLSX)

S2 Data Profile chromatograms and reports of the spitting cobra venoms analyzed through RP-HPLC. Conditions and methods are described in the Material and Methods section. (RAR)

S3 Data Profile chromatograms and reports of the non-spitting cobra venoms analyzed through RP-HPLC. Conditions and methods are described in the Material and Methods section. (RAR)

S4 Data Partial proteomic analyses of the cobra venoms assessed. The partial identification was conducted using a gel digestion of the protein bands of interest and submitted to MS/MS analysis. (XLSX)

## References

[1] Pyron RA, Burbrink FT, Colli GR, de Oca AN, Vitt LJ, Kuczynski CA, et al. The phylogeny of advanced snakes (Colubroidea), with discovery of a new subfamily and comparison of support methods for likelihood trees. Mol Phylogenet Evol. 2011;58(2):329–42. doi: 10.1016/j.ympev.2010.11.006. Epub 2010 Nov 11. . 21074626

[2] WHO (World Health Organization). Guidelines for the production, control, and regulation of snake antivenom immunoglobulins. WHO: Geneva; 2016.

[3] Wüster W, Crookes S, Ineich I, Mané Y, Pook CE, Trape J-F, et al. The phylogeny of cobras inferred from mitochondrial DNA sequences: evolution of venom spitting and the phylogeography of the African spitting cobras (Serpentes: Elapidae: *Naja nigricollis* complex). Mol. Phylogen. Evol. 2007;437–453. doi: 10.1016/j.ympev.2007.07.021 17870616

[4] Malih I, Ahmad Rusmili MR, Tee TY, Saile R, Ghalim N, Othman I. Proteomic analysis of moroccan cobra *Naja haje legionis* venom using tandem mass spectrometry. J. Proteome. 2014;96:240–252, 10.1016/j.jprot.2013.11.012 24269350

[5] Lauridsen LP, Laustsen AH, Lomonte B, Gutiérrez JM. Exploring the venom of the forest cobra snake: Toxicovenomics and antivenom profiling of *Naja melanoleuca*. J. Proteomics. 2017;150:98–108, doi: 10.1016/j.jprot.2016.08.024 27593527

[6] Silva-de-Franca F, Villas-Boas IM, Serrano SMdT, Cogliati B, Chudzinski SAdA, Lopes PH, et al. *Naja annulifera* Snake: New insights into the venom components and pathogenesis of envenomation. PLoS Negl Trop Dis. 2019;13(1):e0007017. 10.1371/journal.pntd.0007017 30657756 PMC6338361

[7] Hus KK, Buczkowicz J, Petrilla V, Petrillová M, Lyskowski A, Legáth J, et al. First Look at the Venom of *Naja ashei*. Molecules. 2018;23:609, doi: 10.3390/molecules23030609 29518026 PMC6017371

[8] Modahl C, Roointan A, Rogers J, Currier K Mackessy SP. Interspecific and intraspecific venom enzymatic variation among cobras (*Naja* sp. And *Ophiophagus Hannah*). Comparative Biochemistry and Physiology, Part C. 2020;232:108743, 10.1016/j.cbpc.2020.108743 32194156

[9] Tan KY, Wong KY, Tan NH, Tan CH. Quantitative Proteomics of *Naja annulifera* (sub-Saharan Snouted Cobra) Venom and Neutralization Activities of two Antivenoms in Africa, Elsevier B.V: 2020, 10.1016/j. ijbiomac.2020.04.173. 32339578

[10] Adamude FA, Dingwoke EJ, Abudakar MS, Ibrahim S, Mohamed G, Klein A, et al. Proteomic analysis of three medically important Nigerian Naja (*Naja haje, Naja katiensis* and *Naja nigricollis*) snake venoms. Toxicon 2021;197:24–32, 10.1016/j.toxicon.2021.03.014 33775665

[11] Sánchez A, Segura Á, Pla D, Munuera J, Villalta M, Quesada-Bernat S, et al. Comparative venomics and preclinical efficacy evaluation of a monospecific *Hemachatus* antivenom towards sub-Saharan Africa cobra venoms. Journal of Proteomics. 2021;240:104196, 10.1016/j.jprot.2021.104196 33775842

[12] Warrell DA, Ormerod LD. Snake venom ophthalmia and blindness caused by the spitting cobra (*Naja nigricollis*) in Nigeria. Am J Trop Med Hyg. 1976;25(3):525–9, doi: 10.4269/ajtmh.1976.25.525 . 1084700

[13] Chu ER, Weinstein SA, White J, Warrell DA. Venom ophthalmia caused by venoms of spitting elapid and other snakes: report of ten cases with review of epidemiology, clinical features, pathophysiology, and management, Toxicon. 2010;56:259–272, 10.1016/j.toxicon.2010.02.023. 20331993

[14] Iddon D, Theakston RDG, Ownby CL. A study of the pathogenesis of local skin necrosis induced by *Naja nigricollis* (spitting cobra) venom using simple histological staining techniques, Toxicon 1987;25:665–672, 10.1016/0041-0101(87)90113-9. 2442855

[15] WHO (World Health Organization). Guidelines for the prevention and clinical management of snakebite in Africa. WHO: Brazzaville; 2010.

[16] Habib AG, Gebi UI, Onyemelukwe GC. Snake bite in Nigeria. Afr. J. Med. Med. Sci. 2001;30:171–178. 14510123

[17] Warrell DA. Snake bite. Lancet. 2010;375(9708):77–88, doi: 10.1016/S0140-6736(09)61754-2. Erratum in: Lancet. 2010;375(9715):640. . 20109866

[18] León G, Sánchez L, Hernández A, Villalta M, Herrera M, Segura A, et al. Immune response towards snake venoms. Inflamm Allergy Drug Targets. 2011;10:381–98, https://doi.org.10.2174/187152811797200605 . 21824081 10.2174/187152811797200605

[19] Casasola A, Ramos-Cerillo B, de Roodt AR, Saucedo AC, Chippaux JP, Alagón A, et al. Paraspecific neutralization of the venom of African species of cobra by an equine antiserum against *Naja melanoleuca*: A comparative study. Toxicon 2009;53:602–608, 10.1016/j.toxicon.2009.01.011 19673073

[20] Wong KY, Tan KY, Tan NH, Tan CH. A Neurotoxic Snake Venom without Phospholipase A2: Proteomics and Cross-Neutralization of the Venom from Senegalese Cobra, *Naja senegalensis* (Subgenus: Uraeus). Toxins (Basel). 2021;13(1):60, doi: 10.3390/toxins13010060 ; PMCID: PMC7828783. 33466660 PMC7828783

[21] Tan CH, Wong KY, Huang LK, Tan KY, Tan NH, Wu WG. Snake Venomics and Antivenomics of Cape Cobra (*Naja nivea*) from South Africa: Insights into Venom Toxicity and Cross-Neutralization Activity. Toxins (Basel). 2022;14(12):860, doi: 10.3390/toxins14120860 ; PMCID: PMC9783313. 36548757 PMC9783313

[22] Bankowski Z& N. Howard-Jones. CIOMS (Council of International Organizations of Medical Sciences). The International Guiding Principles for Biomedical Research Involving Animals. Geneva. 1986.

[23] Lomonte B, Calvete JJ. Strategies in ’snake venomics’ aiming at an integrative view of compositional, functional, and immunological characteristics of venoms. J Venom Anim Toxins Incl Trop Dis. 2017;28:23–6, https://doi.org.10.1186/s40409-017-0117-8 . 28465677 10.1186/s40409-017-0117-8 PMC5408369

[24] Herrera C, Bolton F, Arias AS, Harrison RA, Gutiérrez JM. Analgesic effect of morphine and tramadol in standard toxicity assays in mice injected with venom of the snake *Bothrops asper*. Toxicon. 2018;154:35–41, doi: 10.1016/j.toxicon.2018.09.012 30268394

[25] Durán G, Solano G, Gómez A, Cordero D, Sánchez A, Villalta M, et al. Assessing a 6-h endpoint observation time in the lethality neutralization assay used to evaluate the preclinical efficacy of snake antivenoms. Toxicon X. 2021;12:100087, doi: 10.1016/j.toxcx.2021.100087 34888521 PMC8634039

[26] Finney DJ. Probit Analysis. Cambridge: Cambridge University Press. 1971.

[27] Gutiérrez JM, Lomonte B, Chaves F, Moreno E, Cerdas L. Pharmacological activities of a toxic phospholipase and isolated from the venom of the snake *Bothrops asper*, Comp. Biochem. Physiol. Part C, Comp. 1986;84:159–164, 10.1016/0742-8413. 2873948

[28] Dole VP. A relation between non-esterified fatty acids in plasma and the metabolism of glucose, J. Clin. Invest. 1956;35:150–154, 10.1172/JCI103259 13286333 PMC438791

[29] Rivel M, Solano D, Herrera M, Vargas M, Villalta M, Segura Á, et al. Pathogenesis of dermonecrosis induced by venom of the spitting cobra, *Naja nigricollis*: an experimental study in mice, Toxicon. 2016;119:171–179, 10.1016/j.toxicon.2016.06.006. 27288896

[30] Talke H, Schubert GE. Enzymatic urea determination in the blood and serum in the Warburg optical test. Klin Wochenschr. 1965;43:174–5, https://doi.org.10.1007/BF01484513 . 14258517 10.1007/BF01484513

[31] Mazzachi BC, Peake MJ, Ehrhardt V. Reference range and method comparison studies for enzymatic and Jaffé creatinine assays in plasma and serum and early morning urine. Clin Lab. 2000;46(1–2):53–5. . 10745982

[32] Rodkey FL. Direct spectrophotometric determination of albumin in human serum. Clin Chem. 1965;11:478–87. . 14277286

[33] Gornall AG, Bardawill CJ, David MM. Determination of serum proteins by means of the Biüret reaction. J. Biol. Chem. 1949;177:751–66. . 18110453

[34] Laemmli U. Cleavage of structural proteins during the assembly of the head of bacteriophage T4. Nature. 1970;227:680–685, https://doi.org.10.1038/227680a0 . 5432063 10.1038/227680a0

[35] Segura Á, Villalta M, Herrera M, León G, Harrison R, Durfa N, et al. Preclinical assessment of the efficacy of a new antivenom (EchiTAb-Plus-ICP) for the treatment of viper envenoming in sub-Saharan Africa. Toxicon. 2010;55(2–3):369–74, https://doi.org.10.1016/j.toxicon.2009.08.010 . 19699756 10.1016/j.toxicon.2009.08.010

[36] Petras D, Sanz L, Segura Á, Herrera M, Villalta M, Solano D, et al. Snake Venomics of African Spitting Cobras: Toxin Composition and Assessment of Congeneric Cross-Reactivity of the Pan-African EchiTAb-Plus-ICP Antivenom by Antivenomics and Neutralization Approaches. J. Proteome Res. 2011;10:1266–1280, doi: 10.1021/pr101040f 21171584

[37] Katali O, Shipingana L, Nyarangó P, Pääkkönen M, Haindongo E, Rennie T, et al. Protein Identification of Venoms of the African Spitting Cobras, *Naja mossambica* and *Naja nigricincta nigricincta*. Toxins (Basel). 2020;12(8):520, doi: 10.3390/toxins12080520 ; PMCID: PMC7472217. 32823821 PMC7472217

[38] Conlon JM, Attoub S, Musale V, Leprince J, Casewell NR, Sanz L, et al. Isolation and characterization of cytotoxic and insulin-releasing components from the venom of the black-necked spitting cobra *Naja nigricollis* (Elapidae). Toxicon X 2020;6 100030, 10.1016/j.toxcx.2020.100030 32550585 PMC7285909

[39] Chwetzoff S, Tsunasawa S, Sakiyama F, Ménez A. Nigexine, a phospholipase A2 from cobra venom with cytotoxic properties not related to esterase activity. Purification, amino acid sequence, and biological properties. J Biol Chem. 1989;264(22):13289–97, . 2753914

[40] Panagides N, Jackson TNW, Ikonomopoulou MP, Arbuckle K, Pretzler R, Yang DC, et al. How the cobra got its flesh-eating venom: cytotoxicity as a defensive innovation and its co-evolution with hooding, aposematic marking, and spitting. Toxins. 2017;9:103, doi: 10.3390/toxins9030103 28335411 PMC5371858

[41] Hewitt CD, Innes DJ, Savory J, Wills MR. Normal biochemical and hematological values in New Zealand white rabbits. Clin Chem. 1989;35(8):1777–9. 2758652

[42] Melillo A. Rabbit Clinical Pathology. Topics in Medicine and Surgery; Journal of Exotic Pet Medicine. 2007;16(3):135–145. 32362792 10.1053/j.jepm.2007.06.002 PMC7185592

[43] Warrell DA, Greenwood BM, Davidson NM, Ormerod LD, Prentice CR. Necrosis, haemorrhage and complement depletion following bites by the spitting cobra (*Naja nigricollis*). Q J Med. 1976 Jan;45(177):1–22. 943796

[44] Gómez A, Sánchez A, Durán G, Cordero D, Segura Á, Vargas M, et al. Intrageneric cross-reactivity of monospecific rabbit antisera against venoms of the medically most important *Bitis* spp. and *Echis* spp. African snakes. PLoS Negl Trop Dis. 2022;16(8):e0010643, 10.1371/journal.pntd.0010643 35960772 PMC9374258

[45] Laustsen AH, Lohse B, Lomonte B, Engmark M, Gutiérrez JM. Selecting key toxins for focused development of elapid snake antivenoms and inhibitors guided by a Toxicity Score. Toxicon. 2015;104:43–45, 10.1016/j.toxicon.2015.07.334 26238171

[46] Sánchez A, Coto J, Segura Á, Vargas M, Solano G, Herrera M, et al. Effect of geographical variation of *Echis ocellatus, Naja nigricollis* and *Bitis arietans* venoms on their neutralization by homologous and heterologous antivenoms. Toxicon. 2015;108:80–83, 10.1016/j.toxicon.2015.10.001 26450770

[47] Calvete JJ. Venomics: integrative venom proteomics and beyond. Biochem. J. 2017;474:611–634, 10.1042/BCJ20160577 28219972

