# Supplementary material for: Intrageneric cross-reactivity of monospecific rabbit antisera against venoms of the medically most important Naja spp. African snakes
Source: PLoS Negl Trop Dis. 2023 Aug 15;17(8):e0011545. doi: 10.1371/journal.pntd.0011545 (PMC10426987; doi:10.1371/journal.pntd.0011545)
Supplement: S1 Table — (DOCX) [file pntd.0011545.s001.docx]

S1 Table. Hematological parameters of rabbit groups immunized with the venoms of the spitting cobras.

| Species | RBC (x10^6^/µl) | MCV (fL) | RDW (%) | RDW (a) | Hct  (%) | Hgb (g/dL) | MCH (pg) | MCHC (%) | WBC (x10^3^/µl) | LYM (x10^6^/µl) | MONO (x10^6^/µl) | GRAN (x10^6^/µl) | LYM (%) | MONO (%) | GRAN (%) | PLT (x10^3^/µl) | Mpv (x10^3^/µl) |
| --- | --- | --- | --- | --- | --- | --- | --- | --- | --- | --- | --- | --- | --- | --- | --- | --- | --- |
| *Spitting cobras* | | | | | | | | | | | | | | | | | |
| *N. ashei* | 5.6 ± 0.8 | 68.8 ± 3.6 | 16.5 ± 1.0 | 44.1 ± 2.2 | 38.2 ± 4.0 | 12.0 ± 1.2 | 21.6 ± 1.1 | 31.4 ± 0.4 | 8.8 ± 1.6 | 3.3 ± 0.8 | 0.9 ± 0.3 | 4.6 ± 0.5 | 37.8 ± 3.1 | 9.9 ± 0.8 | 52.3 ± 3.5 | 421.5 ± 75.6 | 3.5 ± 0.3 |
| *N. katiensis* | 6.5 ± 0.5 | 66.4 ± 3.5 | 17.1 ± 1.3 | 43.2 ± 3.1 | 42.9 ± 3.0 | 13.5 ± 0.8 | 20.9 ± 0.9 | 31.5 ± 0.7 | 10.1 ± 1.8 | 4.0 ± 1.0 | 1.1 ± 0.2 | 5.1 ± 0.8 | 39.2 ± 4.8 | 9.9 ± 0.4 | 51.0 ± 5.0 | 385.5 ± 82.6 | 3.7 ± 0.4 |
| *N. mossambica* | 6.1 ± 0.7 | 65.9 ± 2.4 | 16.3 ±0.9 | 41.4 ± 1.0 | 39.9 ± 3.2 | 12.7 ± 1.3 | 21.0 ± 0.4 | 31.9 ± 0.8 | 8.8 ± 1.3 | 3.7 ± 0.6 | 0.9 ± 0.1 | 4.3 ± 0.7 | 41.8 ± 2.4 | 9.1 ± 1.0 | 49.1 ± 2.1 | 331.5 ± 91.5 | 3.4 ± 0.2 |
| *N. nigricincta* | 6.8 ± 0.3 | 65.2 ± 3.1 | 15.5 ± 0.9 | 39.4 ± 1.5 | 44.0 ± 1.7 | 13.9 ± 0.5 | 20.6 ± 0.7 | 31.6 ± 0.6 | 8.3 ± 1.5 | 3.4 ± 0.7 | 0.8 ± 0.2 | 4.1 ± 0.8 | 41.5 ± 2.4 | 8.6 ± 1.1 | 50.0 ± 3.1 | 281.3 ± 80.2 | 4.3 ± 0.3 |
| *N. nigricollis* | 6.1 ± 0.5 | 66.1 ± 1.2 | 17.0 ± 1.9 | 42.0 ± 2.7 | 40.6 ± 2.5 | 12.9 ± 0.9 | 21.1 ± 0.3 | 31.9 ± 0.6 | 9.6 ± 1.9 | 3.6 ± 0.3 | 0.9 ± 0.2 | 5.1 ± 1.7 | 38.6 ± 8.0 | 8.5 ± 1.2 | 52.9 ± 8.2 | 440.8 ± 71.6 | 3.8 ± 0.5 |
| *Non-spitting cobras* | | | | | | | | | | | | | | | | | |
| *N. anchietae* | 5.7 ± 0.9 | 67.9 ± 1.2 | 15.6 ± 0.5 | 41.4 ± 2.3 | 40.1 ± 3.8 | 13.0 ± 1.4 | 22.0 ± 0.9 | 32.4 ± 0.7 | 9.4 ± 1.6 | 2.5 ± 1.5 | 0.9 ± 0.2 | 6.0 ± 1.4 | 26.4 ± 13.0 | 9.2 ± 1.9 | 64.4 ± 12.6 | 364.0 ± 30.0 | 3.7 ± 0.5 |
| *N. annulifera* | 6.9 ± 0.5 | 64.0 ± 3.6 | 16.5 ± 0.7 | 40.1 ± 3.4 | 44.3 ± 1.9 | 14.2 ± 0.5 | 20.6 ± 1.1 | 32.1 ± 0.5 | 9.5 ± 2.0 | 2.6 ± 0.6 | 1.0 ± 0.2 | 5.9 ± 1.3 | 27.3 ± 2.8 | 10.5 ± 0.4 | 62.3 ± 2.9 | 403.0 ± 73.3 | 3.8 ± 0.2 |
| *N. haje* | 6.5 ± 1.1 | 68.4 ± 2.5 | 15.2 ± 0.3 | 41.5 ± 1.9 | 44.4 ± 6.5 | 14.1 ± 1.8 | 21.8 ± 1.2 | 31.8 ± 0.6 | 8.3 ± 0.9 | 2.6 ± 1.0 | 0.8 ± 0.3 | 4.8 ± 0.5 | 31.5 ± 10.0 | 9.3 ± 1.9 | 59.2 ± 11.4 | 326.7 ± 85.7 | 3.9 ± 0.8 |
| *N. melanoleuca* | 6.2 ± 0.4 | 66.2 ± 3.2 | 15.5 ± 0.7 | 40.3 ± 2.9 | 41.3 ± 0.8 | 13.2 ± 0.4 | 21.1 ± 0.9 | 31.9 ± 0.3 | 7.8 ± 2.4 | 2.5 ± 0.7 | 0.7 ± 0.1 | 4.6 ± 2.4 | 33.6 ± 11.1 | 8.6 ± 1.5 | 57.8 ± 12.4 | 378.5 ± 67.5 | 3.8 ± 0.3 |
| *N. nivea* | 5.8 ± 0.2 | 66.9 ± 1.8 | 15.4 ± 1.3 | 40.7 ± 2.6 | 38.5 ± 1.4 | 12.2 ± 0.3 | 21.3 ± 0.6 | 31.8 ± 0.4 | 9.6 ± 1.8 | 2.2 ± 0.6 | 0.8 ± 0.3 | 6.7 ± 1.2 | 22.3 ± 3.3 | 8.1 ± 2.2 | 69.5 ± 5.0 | 395.3 ± 112.8 | 3.4 ± 0.3 |
| *N. senegalensis* | 5.8 ± 0.2 | 66.9 ± 1.8 | 15.4 ± 1.3 | 40.7 ± 2.6 | 38.5 ± 1.4 | 12.2 ± 0.3 | 21.3 ± 0.6 | 31.8 ± 0.4 | 9.6 ± 1.8 | 2.2 ± 0.6 | 0.8 ± 0.3 | 6.7 ± 1.2 | 22.3 ± 3.3 | 8.1 ± 2.2 | 69.5 ± 5.0 | 395.3 ± 112.8 | 3.4 ± 0.3 |
| Control | 6.0 ± 1.5 | 62.1 ± 5.2 | 18.1 ± 2.7 | 41.1 ± 7.3 | 36.6 ± 6.8 | 12.0 ± 2.4 | 20.2 ± 1.4 | 32.6 ± 0.6 | 6.1 ± 1.8 | 2.1 ± 0.7 | 0.7 ± 0.2 | 3.4 ± 1.1 | 36.2 ± 7.2 | 9.4 ± 1.6 | 54.4 ± 5.9 | 452.8 ± 136.1 | 4.0 ± 0.3 |

No significant differences were observed in any of the parameters when compared to values of control rabbits.

**RBC**: Red Blood Cells; **MCV**: Mean Corpuscular Volume; **RDW**: Red blood cell Distribution Width; **Hct**: Hematocrit; **Hgb**: hemoglobin; **MCH**: Mean Corpuscular Hemoglobin; **MCHC**: Mean Corpuscular Hemoglobin Concentration; **WBC**: White Blood Cells; **LYM**: Lymphocytes; **MONO**: Monocytes; **GRAN**: Granulocytes; **PLT**: Platelets; **MPV**: Mean Platelet Volume.
