# Supplementary material for: Intrageneric cross-reactivity of monospecific rabbit antisera against venoms of the medically most important Naja spp. African snakes
Source: PLoS Negl Trop Dis. 2023 Aug 15;17(8):e0011545. doi: 10.1371/journal.pntd.0011545 (PMC10426987; doi:10.1371/journal.pntd.0011545)
Supplement: S2 Table — (DOCX) [file pntd.0011545.s002.docx]

S2 Table. Biochemical parameters of rabbit groups immunized with spitting and non-spitting cobra venoms. Values are compared to the Control group and range reference values are provided. Results are presented as mean ± SD.

| SPECIES | | ALT  (U/L) | AST  (U/L) | ALP  (U/L) | CK  (µmol/l) | UREA  (mmol/l) | Creatinine  (µmol/l) | TP  (g/l) | Albumin  (g/l) |
| --- | --- | --- | --- | --- | --- | --- | --- | --- | --- |
| *Spitting Cobras* | | | | | | | | | |
| *N. ashei* | | 67 ± 22 | 80 ± 44 | 90 ± 11 | 2375 ± 832 | 6 ± 1 | 90 ± 11 | 66 ± 3 | 42 ± 1 |
| *N. katiensis* | | 61 ± 15 | 144 ± 77* | 126 ± 17 | 2484 ± 1032 | 6 ± 2 | 100 ± 12 | 69 ± 4 | 44 ± 1 |
| *N. mossambica* | | 70 ± 30 | 129 ± 39* | 166 ± 32* | 2492 ± 348 | 6 ± 1 | 81 ± 8 | 64 ± 5 | 42 ± 4 |
| *N. nigricincta* | | 54 ± 22 | 91 ± 13 | 182 ± 16* | 2044 ± 1435 | 6 ± 1 | 88 ± 15 | 66 ± 3 | 43 ± 2 |
| *N. nigricollis* | | 54 ± 23 | 79 ± 33 | 157 ± 34* | 1920 ± 314 | 6 ± 1 | 93 ± 12 | 66 ± 4 | 41 ± 2 |
| *Non-spitting Cobras* | | | | | | | | | |
| *N. anchietae* | | 50 ± 9 | 24 ± 7 | 93 ± 58 | 1197 ± 260 | 7 ± 2 | 65 ± 11 | 71 ± 2* | 39 ± 7 |
| *N. annulifera* | | 55 ± 18 | 32 ± 14 | 105 ± 21 | 1090 ± 455 | 7 ± 2 | 79 ± 11 | 72 ± 2* | 42 ± 5 |
| *N. haje* | | 53 ± 12 | 49 ± 52 | 104 ± 42 | 1330 ± 493 | 7 ± 1 | 76 ± 23 | 70 ± 8 | 41 ± 7 |
| *N. melanoleuca* | | 60 ± 15 | 26 ± 4 | 102 ± 37 | 1351 ± 596 | 9 ± 1* | 88 ± 11 | 69 ± 7 | 43 ± 4 |
| *N. nivea* | | 46 ± 5 | 24 ± 1 | 62 ± 25 | 1245 ± 380 | 8 ± 1 | 93 ± 21 | 67 ± 4 | 37 ± 7 |
| *N. senegalensis* | | 47 ± 10 | 35 ± 26 | 70 ± 7 | 1461 ± 663 | 9 ± 3* | 92 ± 18 | 70 ± 4 | 41 ± 5 |
| Control | | 49 ± 9 | 24 ± 6 | 80 ± 53 | 1137 ± 654 | 6 ± 1 | 82 ± 3 | 60 ± 8 | 38 ± 4 |
| Reference | LL | 14 | 14 | 4 | 1.63 | 5.35 | 44.2 | 54 | 25 |
|  | UL | 80 | 113 | 70 | 559.53 | 17.85 | 229.84 | 75 | 50 |

*Significant different (*p*< 0.05) when compared to the control group. **ALT**: alanine aminotransferase, **AST**: aspartate aminotransferase, **ALP**: alkaline phosphatase, **CK**: creatine kinase, **TP**: total protein, LL: lower limit, UL: upper limit
